# A Review of Biomechanical and Physiological Effects of Using Poles in Sports

**DOI:** 10.3390/bioengineering10040497

**Published:** 2023-04-21

**Authors:** Maximilian Saller, Niko Nagengast, Michael Frisch, Franz Konstantin Fuss

**Affiliations:** Chair of Biomechanics, Faculty of Engineering Science, University of Bayreuth, 95447 Bayreuth, Germany

**Keywords:** poles, skiing, walking, running, biomechanics, physiological parameters, coordination, pole properties

## Abstract

The use of poles in sports, to support propulsion, is an integral and inherent component of some sports disciplines such as skiing (cross-country and roller), Nordic walking, and trail running. The aim of this review is to summarize the current state-of-the-art of literature on multiple influencing factors of poles in terms of biomechanical and physiological effects. We evaluated publications in the subfields of biomechanics, physiology, coordination, and pole properties. Plantar pressure and ground reaction forces decreased with the use of poles in all included studies. The upper body and trunk muscles were more active. The lower body muscles were either less active or no different from walking without poles. The use of poles led to a higher oxygen consumption (VO2) without increasing the level of perceived exertion (RPE). Furthermore, the heart rate (HR) tended to be higher. Longer poles reduced the VO2 and provided a longer thrust phase and greater propulsive impulse. The mass of the poles showed no major influence on VO2, RPE, or HR. Solely the activity of the biceps brachii increased with the pole mass.

## 1. Introduction

In cross-country skiing and Nordic walking, poles are a common piece of equipment to help propulsion and engage the whole body. In recent years, professional trail runners have begun to use poles to gain a competitive edge. While the increasing number of runners using poles suggests a beneficial influence of poles, no study summarising the effects of poles could be found in the existing literature.

Poles are, in the context of this review, specific sport equipment used for assisted propulsion for various sports such as cross-country skiing and roller-skiing, Nordic walking, trail running, and rollerblading. A pole consists of different parts, from top to bottom: the grip, the strap, the shaft, the basket, and the tip. The ergonomically shaped grip serves to transfer the force from the arm to the pole, while the strap secures the pole to the hand. The shaft is usually lightweight, manufactured from aluminium or fibre composites (fibreglass, carbon fibres). The tip serves to transfer the force from the pole to the ground, while the basket limits the penetration of the tip into the ground.

The aim of this review is to present the current state of science in relation to pole usage. Alpine skiing in general has not been included in this review due to different pole use or pole types. This review focuses on biomechanics, physiological parameters, coordinative abilities, and the properties of the pole. 

In the biomechanics section of this review, it is shown how the forces on the body change when using poles, specifically as far as the ground reaction forces are concerned. Physiological parameters such as oxygen uptake, heart rate, and perceived exertion are explored as to whether the use of poles has a performance-enhancing effect. The length of the pole is an important factor in influencing the maximum benefit for the user, be it for cross-country skiing in the classic case or for skating technique, trail running, hiking, or Nordic walking. The pole supports the technique of the athlete, improves stability, minimises the fall risk, and optimally transfers the power. 

## 2. Methods

### 2.1. Search Strategy

In order to reliably structure the gathered information in this systematic review, the guidelines and recommendations contained in the PRISMA statement [1,2] were followed. The search was conducted from online electronic databases such as Google Scholar and PubMed, as well as from offline (if not available online) book and journal collections in the field of sports engineering and technology to identify articles between 1990 and 2022. The following sources were included: journals—*Sports Engineering*, *Sports Technology*, *Journal of Sports Engineering and Technology* (proceedings of the Institution of Mechanical Engineers, Part P); conferences—proceedings of the ISEA (International Sports Engineering Association) conferences “The Engineering of Sport”; proceedings of the APCST (Asia-Pacific Conference on Sport Technology) conferences “The Impact of Technology on Sport”. The search term combinations were: 

running OR walking OR skiing OR skating OR blading

AND 

with poles OR pole use OR pole weight OR pole mass OR pole length OR biomechanics OR physiological response OR coordination.

### 2.2. Study Selection

First, the findings were filtered by screening the titles and abstracts. Potentially relevant articles were saved and considered for a full text review. Studies were only included if the following criteria were fulfilled:

1. Published in English;
2. Studies that included two handheld poles to support propulsion;
3. Studies that exclusively included healthy and non-pathological participants;
4. Studies that included subjects averaging younger than 60 years of age.

### 2.3. Data Extraction and Analysis

If the study matched all inclusion criteria, the entire text was analysed, and the results tabulated. The results were divided into different fields such as biomechanics, physiological parameters, coordinative abilities, and properties of the pole. When comparing the key outcomes, the level of statistical significance was recorded (*p* < 0.05). 

## 3. Results

### 3.1. Search Results

**Figure 1:**
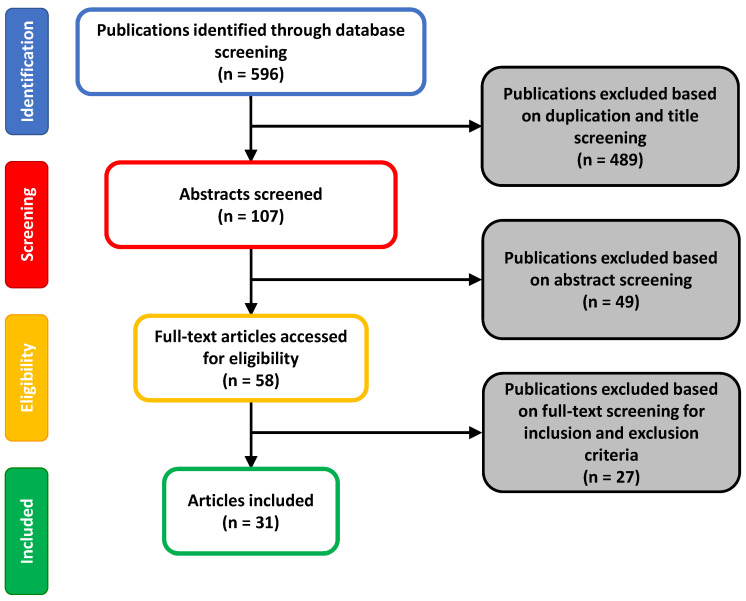
Strategy of the literature review process.

The process of study design, screening, and selection followed the recommendations by [1,2] and is presented by a flow diagram in Figure 1. Through initial database research, a total of 595 potentially relevant publications were identified. After title screening and removal of duplicates, 106 articles remained for abstract screening. Subsequently, 49 studies were excluded because they did not meet the inclusion criteria. 57 full-text articles were assessed for eligibility. As a result, a total of 31 studies were included in this review: 11 publications in the subfield of biomechanics, 10 for physiological parameters, 2 for coordinative abilities, and 8 related to pole properties. In total, 430 subjects participated in the included studies.

### 3.2. Biomechanics

Table 1 shows the study and participant details, aim, sports, environment, and main outcomes of all eleven included studies that focused on biomechanics while using poles. 

Six studies investigated the change in forces acting on the body when using poles during running and walking. Three articles measured the changes of plantar pressure. The investigation of Daviaux et al. [3] was conducted under field conditions on a loop track while trail running 6° downhill, 9° uphill, and on level terrain with and without poles. Plantar pressure was measured with smart insoles (Pedar System, Novel Electronics, Munich, Germany) and the speed was kept at 3.2 m/s [3]. When using poles on level terrain, the mean values of force decreased by 11% and the total force–time integral (impulse) dropped by 4.9% [3]. This decrease in force was primarily observed in the forefoot with a decrease in the medial-forefoot impulse of 12.6% [3]. While downhill conditions reduced the impulse of the medial heel force by 14.2% and the contact time by 13.5%, no changes were found in uphill conditions [3] The second study measured plantar pressures with smart insoles (equally Pedar) during Nordic running over flat grass surfaces at a pace of 3.85 m/s. The authors measured a decrease in average maximum force of 4.8% at the left and 5.3% at the right foot while running with poles [10]. Contrary to [3], ref. [10] reported significant differences of the average maximal force in the forefoot on the one hand and, on the other hand, hardly any changes in the average maximal force in the midfoot and the heel area [10]. The third study [7] measured the plantar pressure when walking barefoot over an indoor walkway (distance = 7.6 m) using a pedobarograph mat. Additionally, the research design differentiated between a two-point and three-point support of the walking style with poles (diagonal and double poling; [7]). This examination found a decrease in pressure under the metatarsal region of the foot. The plantar pressure decreased by 18% in the two-point support style (diagonal) and by 24% in the three-point support style (double poling), respectively [7]. The impulse at the metatarsal region dropped by 15% in the two-point and 19% in the three-point style. No differences in pressure or impulse were found at the heel or the hallux [7]. The results of [3,7,10] cannot be compared directly, as they report different output parameters such as mean force, maximum force, impulse, and plantar pressure. 

Three studies measured the ground reaction forces when using poles. All studies were carried out in controlled environments over 6-m [4], 12-m [5], and 10-m [13] walkways instrumented with force plates. Ref. [4] stated a decrease in vertical ground reaction forces of 2.9% with an unsupervised use of poles. This reduction was also driven to 4.4% by advising the subjects to angle the tip of the pole backwards. When turning the pole forward, this reduction reached a 3.3% decrease in vertical ground reaction forces [4]. 

The second study subdivided the ground reaction force into initial, mid, and terminal stance phases. During the initial and terminal stance phase, an increase in ground reaction force was measured [5]. A decrease in ground reaction force was observed during the mid-stance [5]. Ref. [13] reported a reduction in the ground reaction force, perpendicular to the ground of at least 8% during Nordic walking.

One study calculated joint moments forces from force-plate data and motion-analysis marker data by using an inverse dynamic approach. Ref. [13] found a significant (*p* < 0.05) reduction in lower limb joint forces (ankle, hip, and knee), while the knee extension moment increased significantly. 

One study measured wrist acceleration to determine the shock produced by poles. Participants walked over artificial turf at different speeds with poles and without poles [11]. Peak wrist acceleration in multiples of gravitational acceleration (*g*) increased as the walking speed did, from 3.3 *g* at 1.39 m/s to 7.6 *g* at 2.36 m/s [11].

Four studies measured surface electromyography (EMG) data of different muscles to analyse muscle activation when walking with poles. Three examinations assessed the upper and lower body muscle activation. The research of [8] measured five lower and four upper body muscles while walking on a treadmill inclined by 20% at a speed of 0.83 m/s. Under this condition, the averaged EMG values of lower limb muscles were 14.8% smaller when walking with poles [8]. This decrease was due to the reduced activation of the following muscles: vastus lateralis (−14 ± 5%), gastrocnemius lateralis (−26 ± 8%), and soleus (−22 ± 6%) [8]. The mean EMG for the latissimus dorsi, biceps brachialis, triceps brachialis, and anterior deltoid increased by about 100%, 50%, 150%, and 70%, respectively [8]. In the study of [6], subjects walked on a level treadmill at 1.27 m/s for 30 min. The authors did not find any difference in the averaged or maximal values of lower limb muscles (rectus femoris, lateral hamstrings, tibialis anterior, and gastrocnemius [6]). In the upper body muscles, the average and maximal values of the biceps brachii, triceps brachii, and deltoid medius increased. In the latissimus dorsi, only the maximal values changed [6]. Ref. [13] measured the activity of seven upper limb muscles, two trunk muscles, and seven lower limb muscles. They reported a significant increase in muscle activity in all upper limb muscles (flexor carpi radialis, biceps, triceps, deltoid, pectoralis major, latissimus, and the upper part of the trapezius), as well as in two trunk muscles (rectus abdominis and erector spinae). In contrast to the results of [8], ref. [13] reported an increased activity of the lower limb muscles, from which the data of the vastus medialis and rectus femoris were significant (*p* < 0.05). This result is validated by the greater knee extension moment calculated by [13], as stated above. Another study examined the trunk muscle activation at different speeds and inclinations of a motorized and instrumented treadmill. Pole walking improved the activation time and the force–time integral (impulse) of external oblique and rectus abdominis muscles at most speeds and degrees of inclination [9]. In level walking, the multifidus muscle impulse increased [9]. The muscle activation time of the erector spinae increased in pole walking at the preferred transition speed, resulting in a greater impulse. The coactivation time of trunk muscles increased in pole walking at a 7% [9]. The results of the four EMG studies confirm the common-sense notion that the entire body is more engaged in the propulsive actions, more than when running or skating without poles. This principle places the propulsion assisted by poles in between bipedal locomotion and speed climbing, where all four limbs and the entire body contribute to running up a climbing wall [14]

Four studies addressed the changes in step and stride characteristics while using poles. Ref. [9] and Pellegrini (2017) carried out their experiments on treadmills. In the investigation from [9], the speed was set to 1.11 m/s in level conditions, while in the study of [12], various speeds at 0° and 7° inclinations were measured. Pellegrini (2017) added poles to the gait of participants and measured a stride frequency reduced by 11%. Additionally, walking with poles resulted in a 10.8% greater energy recovery through the pendular swing of the arms [12]. The other investigation conducted on a treadmill showed an increase in stride length for all combinations of speeds and grades during pole usage [9]. In addition, with higher speed, the stride length increased [9]. Two studies analysed various gait parameters while walking over a 6-m [4] and 12-m [5] walkway. Ref. [4] found that poles allow a faster walking speed (+3.6%), longer stride length (+6.2%), and longer stance time (+2.3% to 3.3%). Ref. [5] measured increased cadence (steps/minute: 104.10 ± 6.32–118.84 ± 5.95), stride length (1.30 ± 0.15 m–1.42 ± 0.10 m), and step length (0.70 ± 0.08 m–0.72 ± 0.06 m). Concurrently, stride time (1.16 ± 0.07 s–1.01 ± 0.05 s) and step time (0.59 ± 0.04 s to 0.51 ± 0.03 s) decreased [5].

### 3.3. Physiology

Table 2 shows the study and participant characteristics, sport, environment, and main outcomes of all ten included studies that assessed endurance parameters while using poles. 

Six of the included studies measured the effects of using poles on oxygen consumption. Four of them were carried out in a laboratory environment on motor-driven treadmills. Ref. [23] measured an increase in VO2 of 37% between Nordic walking and walking on a flat surface at 1.53 m/s. Ref. [24] noticed a 12% increase in oxygen consumption (+2.2 mL·min^−1^·kg^−1^) when walking with Exerstriders on a treadmill at 1.86 m/s for 30 min. Ref. [22] also found an increased VO2 of 22.3% at level grade with a speed of 1.11 m/s. Additionally, the authors examined the effects of pole usage on a 15% incline where VO2 was increased by 6.9% compared to normal walking [22]. Ref. [16] investigated the influence of pole usage in uphill, downhill, and level conditions. Contrary to the previous studies, this investigation only noticed significant (*p* < 0.05) higher VO2 values for the downhill condition at a 15% decline, whereas no differences were found for level or uphill walking [16]. 

Two studies were carried out outdoors under field conditions. The first of these two was realized on a 1.25 km track with varying inclines from 0 to <10% [15]. Oxygen consumption on this outdoor trail was increased significantly (*p* < 0.05) at any given incline [15]. The second investigation conducted under field conditions was carried out on a 1.68 km uphill trail with an average incline of 12.6% [20]. During the first study, this trial also detected an increase in VO2 with the use of poles [20].

Ref. [17] calculated the vertical cost of transport at different inclines based on VO2 values. Therefore, VO2 was measured at seven different inclines and converted into the vertical cost of transport. The inclines measured on a treadmill were 10.1°, 15.5°, 19.8°, 25.4°, 29.8°, 35.5°, and 38.9° [17]. On inclines of 25.4°, 29.8°, and 35.5°, it was shown that walking with poles was more effective than walking normally [17]. 

Both the laboratory and field tests showed an increase in VO2 uptake during pole use at an intermediate inclination (<16°) of the (artificial) path. These results did not depend on different speeds or distances of the different study designs. Only [17] demonstrated a potential ‘sweet spot’ (approximately 20–36°) of incline at which pole use may reduce the oxygen uptake. Further research should include verification of these ‘sweet spots’ in both inclined and sloping terrains.

Since all sports in which pole usage plays a role (running, trekking, Nordic walking, and cross-country skiing) rely primarily on aerobic metabolism (final sprints in competition excluded; [25]), beneficial symbioses or correlations between the different disciplines may be of interest. Specific metabolic-based training patterns for pole use might be imperative to develop and correlate.

Regarding the sample parameters, the results do not correlate with the selection of the population. From different levels of ability (beginners to mountain running and Nordic walking instructors) to female, male, or gender combinations and different age groups (20–50 years), no significant differences were observed in the main results.

Nine included studies mentioned effects on heart rate. Four authors observed an increase in heart rate with the use of poles. Measurements by [19] were performed on a level tartan track at an individual speed below the anaerobic threshold at about 60–80% of VO2max. While running with poles, the heart rate increased by 4.5% [19]. The participants of [23] walked on a motor-driven treadmill at 1.53 m/s. An average increase of 23 beats per minute was encountered while walking with poles [23]. The participants of [24] walked on a motor-driven treadmill at 1.86 m/s and experienced an average increase of 12 beats per minute while walking with poles for 30 min. The fourth study regarding heart rate was performed at a self-selected speed on an outdoor track with varying inclines from 0 to <10% [15]. The increase in heart rate only refers to the pole versus non-pole condition. No interaction between heart rate, pole usage, and inclination could be detected [15]. 

Opposed to these four findings, the other five investigations could not find any dependencies on the heart rate when using poles. Refs. [16,17] realized measurements on indoor treadmills. Both had trials of level and inclined walking. While ref. [17] had subjects perform at a speed set of 80% of the maximal velocity of each participant, the participants from [16] walked at self-selected speeds. The other three studies measured parameters in field conditions. Two of these collected data only during uphill hiking [20,21]; however, ref. [18] also measured the heart rate during descents. Subjects in the investigation of [21] were advised to exert maximal effort while participants from [18,20] walked at a self-selected pace. 

Seven included articles measured the rating of perceived exertion (RPE) of subjects. None of these found an increase in RPE while using poles. Four studies could not find any changes in RPE by using poles. Refs. [15,20,21] carried out their research in field conditions on tracks with level and inclined sections. Refs. [16,24] investigated walking on a levelled treadmill. Ref. [16] included an additional 15% decline. Ref. [18] described that RPE dropped when using poles during inclined walking. Ref. [18] investigated RPE under field conditions and could only find a decrease in RPE during ascents, while no difference was detected during descents. Ref. [18] found that RPE showed significantly lower results only during treadmill slopes of 15.5° (*p* = 0.002), 19.8° (*p* = 0.002), 29.8° (*p*  =  0.005), 35.5° (*p* = 0.007), and 38.9° (*p* = 0.001).

Three included articles measured blood lactate levels. Two investigations tested walking conditions with poles. The first one examined participants on a treadmill at level running and different inclines [17]. The second one reviewed participants at a maximum effort mountain ascent [21]. No differences in blood lactate levels could be found during either protocol. Ref. [19] conducted running with poles on a tartan track at a speed of 60–80% of VO2max. This examination identified an increase in lactate levels of 88.3% when using poles for running compared to running without [19]. 

Four included studies analysed respiratory parameters. Ref. [15] encountered an increase in ventilation for all downhill, flat, and uphill slopes when using poles. The ventilation volume was measured in litres per minute, which increased in all pole conditions [15]. The second study noticed an elevation in minute ventilation for downhill and level trails. This examination was conducted on a treadmill at a self-selected pace [16]. Additionally, the breathing frequency accelerated when using poles, in absolute values at every grade, but only significantly (*p* < 0.05) in downhill conditions [16]. During uphill conditions, the tidal volume decreased by 9% while using poles [16]. Ref. [24] reported an increase in the respiratory exchange ratio (+0.04) when walking with poles. Contrary to previous findings, [17] did not report any differences in tidal volume at any incline. In addition, minute ventilation was mostly lower with poles but not statistically significant (*p* = 0.17; [17]). 

One study reported that the total caloric expenditure was significantly greater during the pole condition by 33 kcal (23.5%; [24]).

One study investigated the effects on pole usage on muscle damage. The results stated that using poles reduces muscle damage after a day of hiking [18]. The authors measured less delayed-onset muscle soreness in the trekking pole group, as well as lower creatine kinase levels. Furthermore, using poles induces a lower isometric strength loss in the lower limbs immediately, as well as after 24 h, following physical stress [18]. 

All listed publications measured simple physiological parameters but lacked statements on more complex relationships. Running economy (RE) is influenced by several biomechanical (gait pattern, kinematics, and kinetics of running) and physiological factors (oxidative muscle capacity, muscle stiffness). An improved RE can resist exercise-induced muscle damage (EIMD) or delayed-onset muscle soreness (DOMS) for longer times [26]. Both [26,27] indicated that plyometric, resistive, or eccentric muscle contractions during exercise are considered an important factor in skeletal muscle damage. In the context of the demanding conditions of the aforementioned sports (Nordic walking, trekking, ultramarathon, cross-country skiing, etc.) in terms of terrain (uphill, downhill) and additional loads (e.g., small backpack for ultramarathons, biathlon rifle, cross-country skis, etc.), the effects on lower limb muscles in terms of EIMD or DOMS can be significant [28]. In addition, the type of exercise (running, walking, cross-country skiing) affects muscle damage and strength loss. The shock spikes during running are responsible for a higher DOMS compared to cross-country [26]. Even though the skeletal muscles fatigue slower and recover faster [29], the higher oxygen uptake and the increased heart rate argue against pole usage, the authors of this review suggest considering the following items where pole use can play a positive role in terms of physiological benefits: (i) the strong eccentric contraction during downhill walking or running can be mitigated; (ii) the main load on the muscles of the lower limbs can be directed to the upper limbs; (iii) the shock spike of foot impacts can be cushioned; and (iv) the peaks of plantar pressure, and thus (v) the muscle fatigue of the legs can be reduced.

Of the four publications in which muscle activations were observed, three ([6,8,13]) compared muscle activation between the lower and upper limbs during pole use. In terms of representative results regarding the total exercise time of the included sports (running, Nordic walking, trekking, cross-country skiing), [13] can be neglected as it only included a 10-m run over a force plate. EMG studies by [6,8] highlight the increased muscle activation of upper limb muscles (latissimus dorsi, biceps brachialis, triceps brachialis, anterior deltoid, triceps brachii, medius deltoid). Considering that muscles fatigue faster and that upper limb muscle groups recover more slowly [29], further studies on the potential optimisation of specific pole-induced RE could be beneficial to reduce DOMS in the upper limbs. When comparing the two studies in terms of study design, [8] incorporated an incline into the test setup leading to an observable decrease in muscle activation of the lower limb muscles. In relation to the level walking method of [6], which did not reveal any significant decrease in the activation of lower limb muscles (rectus femoris, lateral hamstrings, tibialis anterior, gastrocnemius), we hypothesise that a significant benefit will be found when studying more complex movement patterns in relation to the slope of the terrain. Further research is required to confirm or reject this hypothesis. 

### 3.4. Coordination

Table 3 shows the study and participant details, aim, sport, environment, and main outcomes of each of the two included articles that focused on coordinative abilities while using poles. 

In the study of [30], the subjects walked on a flat treadmill at 1.53 m/s with and without poles. The inclusion of poles enhanced the mediolateral stability while anterior–posterior stability remained unchanged [30]. The increase in mediolateral stability occurred alongside a greater stability in trunk coordination [30]. The second study analysed the muscular synergy patterns of subjects walking on a treadmill at 1.3 m/s with and without poles [31]. The authors found five main muscle synergies in the gait pattern of walking without poles and of Nordic walking. Between walking and Nordic walking, just one muscle synergy responsible for upper limb propulsion showed differences in the gait cycle. The gait pattern is characterized by a reduced activation of the tibialis anterior and increased activation of the latissimus dorsi, posterior deltoid, and triceps brachii [31]. The other four synergies—mainly describing lower limb activation—did not show substantial differences when using poles [31].

### 3.5. Properties of the Poles 

Table 4 shows the study and participant details, aim, sport, environment, and main outcomes of all eight included studies that focused on pole length and pole mass. 

#### 3.5.1. Pole Length 

In total, five included studies provided evidence of the pole length’s influence. 

Four of these examined the oxygen consumption when using different pole lengths. In the investigation of [32], male cross-country skiers conducted one 1000 m maximal effort and three submaximal bouts at 3.0, 3.5, and 4.0 m/s at a 2.5° incline. The participating skiers used poles of self-selected length (84 ± 1% of body height) and poles 7.5-cm longer than the standard length [32]. During the submaximal bouts, the use of longer poles induced a decrease in O2 cost at all speeds of an average of 2.7 ± 0.7% [32]. 

The second study examined cross-country skiers on a treadmill in two conditions (1.7° inclination at 4.5 m/s, and 4.5° at 2.5 m/s) with self-selected poles (84 ± 1% of body height, SS; [33]). Additionally, subjects were given poles five centimetres shorter and poles five and ten centimetres longer than SS (Carlsen et al., 2018). In both conditions, the longest poles (SS + 10 cm) led to the lowest O2 cost compared to all other lengths [33]. Compared to SS, SS + 10 cm decreased the O2 cost by 2.1  ±  1.1% in a lower inclination and by 4.0  ±  1.0% in a greater inclination [33]. There was no difference between SS − 5 cm, SS, and SS + 5 cm in the lower inclination (1.7° at 4.5 m/s). At the greater inclination (4.5° at 2.5 m/s), the O2 cost increased as length decreased, with SS − 5cm demanding the highest O2 cost [33]. 

The study of [38] conducted classic roller skiing on a 2° inclined treadmill at a speed under the participants’ anaerobic threshold (90% of anaerobic threshold). The subjects selected the poles from a set of seven different pole lengths. The participants skied with self-selected poles (averaged at 87% of body height), as well as shorter and longer ones. The self-selected poles ranged from 86% to 89% of the subject’s body height. The smallest ratio of pole length to body height was 77%, while the greatest length to body height ratio was 98% [38]. This study also found a lower oxygen uptake when using longer poles [38].

In the fourth study, subjects performed Nordic walking with self-selected poles (67.6 ± 0.6% of body height) and poles 7.5-cm shorter than self-selected ones [35]. A treadmill was set to inclinations of 12° uphill, horizontal, and 12° downhill [35].Each participant selected his/her own speed. No differences between pole lengths were detected for horizontal and downhill walking. In inclined conditions, the 7.5-cm shorter poles resulted in a higher O2 consumption of 3.2 ± 1.0% [35].

Two studies considered the effects on heart rate and RPE. Ref. [32] found no differences in heart rate or RPE, in either the submaximal or maximal bout, when using longer poles. Ref. [33] also found no effect of pole length on heart rate or RPE. Differences in incline were the only factor that affected heart rate and RPE [33]. 

Two studies reported kinematic changes when using different pole lengths. In the first, the participants used self-selected poles, and poles longer and shorter by 7.5 cm than the self-selected ones (means: 82, 86, and 78% of body height; [37]). Subjects performed double poling on roller skis over a level force plate after gaining speed on a 6.3-m downhill slope. From the shortest to the longest pole, the duration of the thrust phase increased by 15% [37]. Longer poles resulted in increased vertical and propulsive anterior–posterior impulses (17% and 8%; [37]). Ref. [38] reported a longer ground contact time and lower poling frequency when using longer poles. The propulsive impulse was positively correlated with the length of the poles [38].

#### 3.5.2. Pole Mass

Three investigations examined the influence on pole mass. In the study by [8], subjects walked on a 20% inclined treadmill at 0.83 m/s using light, medium, and heavy poles (240 g, 300 g, and 360 g). No correlation between pole mass and VO2 was detected. With medium and heavy poles, the biceps brachii activation (EMG) was expectedly greater than that found using light poles [8]. In terms of the recovery time, the activation of the triceps brachii and biceps brachii was greater with heavy poles. The activation of the anterior deltoid was greater with a lower position of the COM (centre of mass) on the pole compared to a higher COM position [8].

The second study conducted a seven-minute 2 kph fast walking activity on a 400 m tartan track without poles, with poles, and with poles with added mass (+0.5 kg, +1 kg, +1.5 kg; [34]). As far as the RPE, VO2, and heart rate are concerned, no correlation between added mass and the physiological parameters was detected [34]. Blood lactate levels were higher when using +1.5 kg poles compared to walking without and with normal poles [34]. The ground contact time of the pole and total propulsion impulse were not affected by the pole mass (Schiffer et al., 2011). The activation of the biceps brachii increased with the heaviest poles (+1.5 kg; [34]) 

In the third study, the participants walked on a levelled treadmill run at 1.67 m/s without poles and with poles, without and with an additional mass of 1 kg [36]. There was no difference in VO2, RPE, or heart rate between the two poling conditions [36]. No EMG value (medial gastrocnemius, tibialis anterior, biceps femoris, rectus femoris, and biceps brachii) was affected by the two conditions with poles [36]. Contrary to poles with average mass (240 g), waking with heavy poles (1 kg) resulted in a greater activation of the biceps brachii [36].

There is evidence that the length of poles in cross-country skiing, Nordic walking, and roller skiing influences oxygen consumption. Using longer poles in different field conditions led to a lower oxygen consumption. However, the heart rate and RPE were not affected at all. A clear positive trend was shown in the effect of the pole length on the dynamics. In the studies by [37,38], it was shown that longer poles led to greater impulses in the anterior–posterior movement and that this propulsive impulse correlates positively with the pole length.

A correlation between the mass of the poles and physiological parameters, such as VO2, HR, or RPE, could not be identified in the available literature. Only the muscle activity of the upper arm muscles (biceps and triceps brachii) increased when the mass of the poles increased. 

## 4. Discussion

This review provides an update and a summary of literature sources assessing the influence of using poles in skiing (cross-country and roller), Nordic walking, and trail running. 

The effect of poles on the biomechanics of the human body reveals that plantar pressure, as well as ground reaction forces, decreased in all studies [3,4,5,7,10,13]. Shocks on the wrist increased when using poles and when walking faster [11]. EMG data measured on trunk und upper body muscles indicated a higher activation with poles [6,8,9]. As far as the lower limb muscles are concerned, one study measured a decreased activation of lower limb muscles with poles [8]; a second study did not find any difference in activation of the lower body muscles [6] between walking with and without poles; while the third study found an increased activity in the quadriceps [13]. The latter EMG result is particularly supported by a concurrent increase in the knee extension moment when using poles, calculated from force-plate and motion-analysis data, despite measuring a reduction in the normal ground reaction force [38]. The reason for this counterintuitive result could be an increased tangential ground reaction force (friction force) at the legs due to acceleration when walking over the force plate. Accelerating on the force plate happens when the walkway is too short (e.g., 5 m before and after the force plate, as seen in the study of [13], instead of walking over the force plate with a constant velocity if the walkway is sufficiently long (e.g., 20+ m). Unfortunately, [13] did not report the magnitude of the tangential ground reaction forces, and therefore the EMG and joint moment data of [38] have to be treated with caution. Differences in the gait patterns were primarily described as an increase in stride length [4,9] (One study reported a greater pendular energy recovery while walking with poles [12].

As far as physiological parameters are concerned, regardless of the testing protocol, all studies reported an increase in VO2 while using poles [15,16,17,20,22,23,24]. No single investigation reported an increase in RPE with the use of poles [15,16,17,18,20,24] (The heart rate was either faster with poles [15,19,23,24] or showed no significant (*p* < 0.05) difference [16,17,18,20,21]. The total caloric expenditure was greater when using poles [24]. Regarding blood lactate levels, walking with poles did not show any difference [17,21], except for one study investigating running with poles that detected a high increase in blood lactate levels [19]. The effect of poles on respiratory parameters varied across different study designs and terrain inclinations [15,16,17]. Nevertheless, the ventilation volume increased in all pole conditions [15]. A study that investigated muscle damage stated a decrease in muscle damage parameters when using poles [18].

Muscle coordination in the lower limbs during Nordic walking can be considered a movement similar to walking—with the only difference being the involvement of the upper limbs for additional propulsion [31]. Therefore, walking with poles is substantially not more complex than conventional walking. These similarities open the field of pole walking to people with less coordinative abilities [31]. One study found an improved mediolateral stability and similar anterior–posterior stability in Nordic walking compared to walking without poles [31]. 

Increased pole length resulted in lower oxygen consumption for inclined conditions [32,33,35,38]. The pole length did not affect the heart rate or RPE [32,33]. With longer poles, the propulsive impulse was greater compared to shorter poles [37,38]. Furthermore, the duration of the thrust phase increased [37,38] (Nilsson et al., 2003; Onasch et al., 2017). The mass of the pole did not have any influence on VO2, RPE, or heart rate [8,34,36]. The deployment of heavier poles increased the activation of the biceps brachii [8,34,36].

These results suggest that including poles in human movement increases the oxygen consumption and thus the energy expenditure, and it is therefore demanding for the metabolism. This increase could be explained by the improvement of the upper body and trunk muscle activation such as the biceps brachii, triceps brachii, and latissimus dorsi. At the same time, the lower limb muscles experience a comparatively smaller but still significant relief through the reduction in the ground reaction forces. 

The increases in the oxygen consumption and in the total caloric expenditure could be a limiting factor for competitive athletes, specifically when running at speeds close to their VO2max. Recreational running or training sessions in general could profit from the higher load on the body without having to increase the speed. Despite the increased load on the body, the perceived rate of exertion (RPE) does not increase when using poles. This is advantageous because athletes can profit from the use of poles without feeling burdened by them. Especially in long-distance races, the mental and subjective perceptions of the athletes play an important role, in addition to physiological parameters. 

Using poles leads to the decrease in ground reaction force, plantar pressure, and reduced indicators of muscle damage, as well as the tendency for decreased lower limb muscle activation. Specifically, if stress is applied over a long period of time, such as in long-distance races or subsequent sessions over several days, the biomechanical effects could help preserve the performance of lower limbs and delay the onset of fatigue. Improved trunk muscle activation and mediolateral stability when using poles renders the movements more stable and controllable.

As far as the pole properties are concerned, the results suggest that pole length has a much greater influence than pole mass. With longer poles, the athlete benefits from a longer thrust phase and propulsive impulse. Additionally, longer poles reduce VO2 and therefore save energy. Adjusting pole length is a simple means for the athlete to improve performance while becoming more efficient. With greater propulsive impulses, the athlete can shift more load to the upper body while off-loading the legs and still generate sufficient acceleration. Pole mass did not have a substantial influence on the measured parameters, except for a greater activation of the biceps brachii. Nonetheless, the pole mass should be kept to an absolute minimum for reducing the overall load on the body. Through the length and mass distribution in the pole, even small differences in mass lead to notable effects [12].

**Figure 2:**
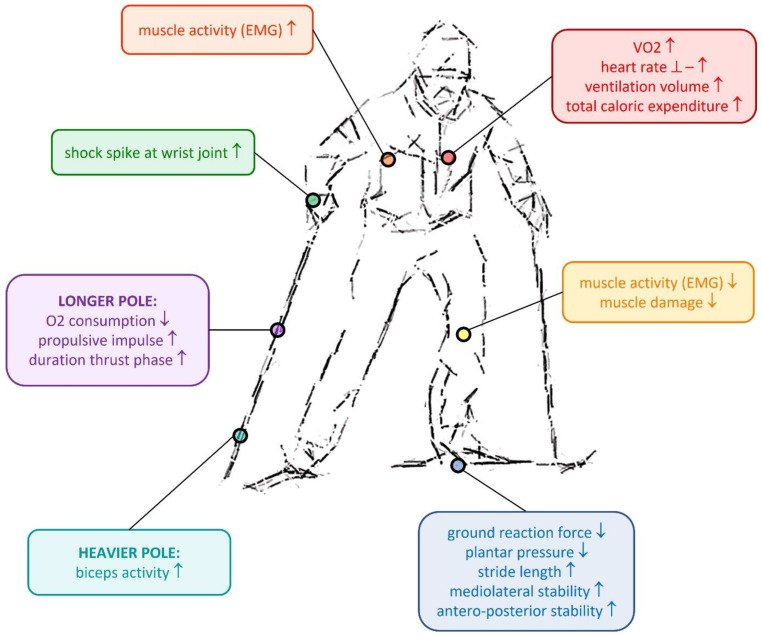
Summary of pole-induced effects on the human body; ↓ = decrease, ↑ = increase, ⟂ = no change.

The key findings of this review are summarised in Figure 2.

This review faces some limitations. The studies included in the review differ substantially in terms of environmental conditions, measured parameters, participants’ characteristics, and experimental procedures. While some investigations addressed the differences in the controlled environment, other test setups were conducted under field conditions. The influence of ground conditions could limit the comparability of these study outcomes. Furthermore, the inclines examined (if any) differed from study to study. The prescribed speed varied between fixed values, fixed self-selected values, or no speed constraint at all. With pole use being a technical skill, the individual skills of subjects could affect the results.

## 5. Conclusions

In conclusion, this review shows that the use of poles comes with advantages and disadvantages. While a certain amount of the load on the body is shifted to the upper limbs, and while forces acting on the lower limbs decrease, the overall metabolic stress on the body increases notably without affecting the perceived exertion. Mediolateral stability is enhanced when using poles and muscle damage in lower limbs is limited, suggesting a lower risk of injury. Nevertheless, further studies are needed to examine the specific responses of the body in sport disciplines relying on the use of poles.

Overall, each literature source on the effect of poles on the human body investigated only a small aspect, be it biomechanics or physiology. However, a comprehensive study, exploring different aspects of pole usage simultaneously, and preferably within the sporting context, is missing. Nevertheless, the laboratory studies are of great value, but outdoor studies are lacking, particularly those supported by wearable measurement technology.

## Figures and Tables

**Table 1:** Biomechanical aspects of pole use.

References	Participant Details	Sport	Aim	Environment	Main Outcomes
[3]	N = 12 Gender: 12 m Mean age: 28.4 ± 8.6	Trail running	Investigation of the effects using poles has on foot–ground interaction during trail running with slopes of varying incline	Outdoor loop track with level, uphill (9°) and downhill (6°) sections	- A decrease in plantar forces even when the running velocity remained constant (force–time integral 4.9% and mean force 11.0%) - Regional analysis revealed a decrease in medial forefoot force–time integral in the pole-use condition (12.6%) - During downhill running, a decrease in medial heel force–time integral (14.2%) and contact time (13.5%) was found with poles
[4]	N = 13 Gender: 8 m/5 f Mean age: 29.5 ± 5.1	Walking	Assessment of whether walking with poles reduces loading to the lower extremities during level overground walking	Controlled 6 m walkway	- Increase in walking speed, stride length, and stance time compared with the no-poles condition. - Decrease in anterior–posterior GRF braking impulse - Decrease in vertical (compressive) knee joint reaction force compared with the no-poles condition - Poling conditions resulted in a decreased average vertical ground reaction force over the no-poles condition. The average force decreased by 2.9% with poles
[5]	N = 30 Gender: 30 m Mean age: NW group: 23.2 ± 4.6 W group: 23.8 ± 3.9	Walking	Investigation of the effects Nordic walking and walking have on spatiotemporal gait parameters and ground reaction force	Controlled 12 m walkway	- Cadence, stride length, and step length were increased - Decrease in stride time, step time, and vertical ground reaction force
[6]	N = 26 Gender: - Mean age: Pole group: 21.3 ± 0.9 Non-pole group: 21.5 ± 0.8	Walking	Assessment of the effect Nordic pole walking has on the EMG activities of upper extremity and lower extremity muscles.	Controlled, treadmill set to 1.27 m/s	- The average and maximum values for the muscle activities in the upper extremity increased in the pole group - The average and maximum values for the muscle activities in the lower extremity did not show any large differences in either the with-pole group or the walking group
[7]	N = 35 Gender: 16 m/19 f Mean age: 27.3	Walking	Investigation of the effectiveness walking with poles has on reducing plantar pressure on the foot	Controlled (7.6 m walkway)	- Decrease in plantar pressure by 18% (diagonal poling) and 24% (double poling) - Decrease mainly in the metatarsal heads with 19% reduction (double poling) and 15% reduction (diagonal poling) - No differences in pressure or impulse were found in the heel or hallux regions
[8]	N = 11 Gender: 11 m Mean age 24.0 ± 4.6	Walking	Investigation of the effect using hiking poles with different inertia has on oxygen cost (VO2) and muscular activity.	Controlled, treadmill set to 0.83 m/s and 20% incline	- Decrease in lower limb muscle mean EMG values by 14.8% with poles - Increase in upper limb muscle mean EMG values
[9]	N = 10 Gender: 10 m Mean age: 28.5 ± 5.6	walking	Observation and comparison of the activity in the trunk muscles during W and PW at different treadmill speeds and grades.	Treadmill at highest transition speed from walking to running	- Increase in stride length - Increase in external oblique and rectus abdominis activation time and force–time integral at most speeds and grades - Increase in trunk muscle coactivation time
[10]	N = 1 Gender: 1 m Age: -	Running	Comparison of the force impact and pressure changes with poles and to evaluate potential changes in foot pressure distribution	Controlled, 100 m grass runway at a pace of 3.85 m/s	- Decrease in average force when using poles - Relocation of the pressure centre to the front part of the foot - Decrease in force in the heel and mid-foot area, while the differences in the forefoot are relatively small
[11]	N = 24 Gender: 12 m/12 f Mean age: 38 ± 13	Nordic walking	Estimation of lower and upper limb injury risks and their dependency on speed	Controlled, on artificial turf at speeds of 1.39, 2.17 and 2.36 m/s	- Peak wrist accelerations from 3.1 up to 7.6 times gravitational acceleration with increasing walking speeds
[12]	N = 8 Gender: 8 m Mean age: 39.6 ± 12.6	Nordic walking	Investigation of the effect NW has on the COM body segments’ movements	Controlled, treadmill at 1.11 m/s	- Decrease in stride frequency by 11%. - Increase in pendular recovery by 10.8% in NW
[13]	N = 9 Gender: 9 m Mean age: 22.9 ± 1.6	Nordic walking	Measurement of ground reaction forces at the feet and poles; calculation of lower limb joint moments and forces; measurement of muscle activity (EMG) of 9 upper limb and 7 lower limb muscles	Controlled, level walking at self-selected speed	- Increase in muscle activity (upper limb, abdominal, spine, quadriceps) - Reduction in ground reaction force at the feet - Increase in knee extension moment - Reduction in joint forces (ankle, knee, hip)

**Table 2:** Physiological aspects of pole use.

References	Participant Details	Sport	Aim	Environment	Main Outcomes
[15]	N = 14 Gender: 14 m Mean age: 22.1 ± 2.1	Hiking	Assessment of the effects of pole usage during fitness walking, as opposed to through-hiking on very loose or demanding terrain, or other activities	A 1.25 km dirt and gravel trail with sections of different inclines	- Increase in VO2 no matter what grade with poles - Increase in ventilation with poles - Increase in heart rate with poles - Pole condition had no influence on RPE
[16]	N = 12 Gender: 5 m/7 f Mean age: 28.4 ± 8.8	Walking	Comparison of physiological responses and RPE during walking exercise trials of different grades carrying or not carrying a backpack loaded to 15% body mass, and with and without hiking poles	Controlled, treadmill	- Increase in ventilation for downhill trails - Increase in VO2 with poles - No influence on heart rate or RPE with the use of poles
[17]	N = 14 Gender: - Mean age: 32.7 ± 6.6	Trail running	Comparison of energy expenditure during uphill walking with (PW) and without (W) poles at different slopes	Controlled, treadmill inclined at 7 different grades	- Decreased vertical cost of transport at 25.4°, 29.8°, and 35.5° - No effect of poles on blood lactate, heart rate, or tidal volume
[18]	N = 37 Gender: 26 m/11 f Mean age = 25 ± 7	Hiking	Examination of the effects trekking poles have on indices of muscle damage	Outdoor track	- No difference in heart rate with poles - Lower RPE in the ascent with pole use - Fewer indices of muscle damage with poles
[19]	N = 12 Gender: 6 m/6 f Mean age: m 18.0 ± 3.8/f 17.3 ± 1.6	Running	Comparison of the load exerted on the organism during running with poles and without poles at the same speed	Controlled, tartan track	- Increase in blood lactate with the use of poles by 88.3% - Increase in average heart rate by 4.5%
[20]	N = 20 Gender:10 m/10 f Mean age 22.7 ± 2.0	Hiking	Estimation of the effects hiking pole use has during continuous uphill hiking	Outdoor, 1.68 km-long uphill trail	- Increase in VO2 with poles - No difference in heart rate, respiratory exchange ratio, and RPE
[21]	N = 15 Gender:7 m/8 f Mean age 29 ± 6	Hiking	Comparison of performance when hiking with and without poles during a maximal effort mountain ascent and differences in physiological responses	Outdoor, 4 km-long uphill hike	- No difference in blood lactate, heart rate, and RPE with poles
[22]	N = 9 Gender: 9 m Mean age 36.8 ± 11.9	Uphill walking	Assessment of differences in muscle activation and physiological responses between Nordic walking and walking in level and uphill conditions	Controlled, treadmill at 0 and 15% incline with given speed at 1.11 m/s	- Increase in VO2 with poles (+22.6% level, 6.9% inclined) and incline
[23]	N = 10 Gender: 5 m/5 f Mean age 37.7 ± 12.0	Nordic walking	Evaluation of the differences in muscle activation and metabolic responses between walking and Nordic walking	Controlled, treadmill at 1.53 m/s	- Increase in VO2 of 37.2% with poles - Increased heart rate with poles (avg. +23 bpm) - Optimal NW technique has the highest influence on muscular and metabolic responses
[24]	N = 10 Gender: 10 f Mean age 23.6 ± 4.0	Walking with Exerstr-iders	Evaluation of the differences in metabolic responses between walking and Exerstriders walking	Controlled, treadmill at 1.86 m/s	- Increase in oxygen consumption (+2.2 mL·min^−1^·kg^−1^), heart rate (+11 bpm on average), respiratory exchange ratio (+0.04), and total caloric expenditure (+33 kcal) with poles

**Table 3:** Coordinative aspects of pole use.

Ref	Participant Characteristics	Sport	Aim	Environment	Main Outcomes
[30]	N = 11 Gender: 6 m/5 f Mean age: 39 (21–54)	Nordic walking	Comparison of stability margins, hip stabilizer muscle activation, and scapular–pelvis coordination between walking and two different pole walking techniques	Controlled, treadmill at 1.39 m/s	- Increased mediolateral stability with poles - Higher stability in trunk muscle coordination - No difference in anterior–posterior stability with poles
[31]	N = 9 Gender: 5 m/4 f Mean age: 39 ± 12 years	Nordic walking	Assessment of whether Nordic walking required a task-specific muscle coordination with respect to conventional walking	Controlled, treadmill at 1.3 m/s	- Muscle synergies in lower limbs stay unaltered with poles - Additional muscle synergy in upper limbs for propulsion with poles including higher activation of the latissimus dorsi, posterior deltoid, and triceps brachii - No increased complexity in movement

**Table 4:** Pole property aspects of pole use.

Ref	Participant Characteristics	Sport	Aim	Environment	Main Outcomes
[32]	N = 9 Gender: 9 m Mean age: 24 ± 3	Cross-country roller skiing	Comparison of self-selected and 7.5 cm-longer poles on performance, O2-cost, and kinematical patterns in the DP technique among competitive cross-country skiers	Controlled, treadmill	- Decrease in VO_2_ at submaximal bouts with longer poles - No difference in heart rate or RPE between pole lengths
[33]	N = 13 Gender: 13 m Mean age: 23 ± 3	Cross-country ski	Comparison of O2-cost and kinematics during double poling with different pole lengths	Controlled, treadmill	- Longer poles induce lower VO2 by 2.1 ± 1.1% in a lower incline and 4.0 ± 1.0% in a higher incline - No difference in heart rate and RPE was found
[34]	N = 12 Gender: 12 f Mean age: 21	Nordic walking	Assessment of the effect of varying pole mass on energy expenditure, upper limb muscle activation, and on forces transmitted to the poles during Nordic walking	Controlled, 400 m tartan track	- Increased VO2 with the use of poles compared to the no-pole condition - Increased mean biceps brachii activation for trails with added mass to poles - No differences in heart rate, RPE, and VO2 were found between different pole mass
[35]	N = 12 Gender: 1 m/11 f Mean age: 50.6 ± 2.4	Nordic walking	Assessment of energy expenditure and self-rated comfort during uphill, horizontal, and downhill Nordic walking with different pole lengths and during ordinary walking	Controlled, treadmill	- Increased VO2 and energy expenditure with shorter poles in inclined conditions - No differences in level and declined grades
[36]	N = 7 Gender: 4 m/3 f Mean age: m 22.5 ± 1.0/f 27 ± 2.9	Nordic walking	Investigation of the effect varying pole mass has on energy expenditure, upper limb, and lower limb muscle activity using an electromyogram during Nordic walking	Controlled, treadmill	- No difference in VO2, RPE, or heart rate between the two poling conditions - Higher biceps brachii activation with heavier poles
[37]	N = 7 Gender: 7 m Mean age: 22	Roller skiing	Investigation of the relationship between thrust phase duration, ground reaction force, and velocity increase with poles	Controlled, laboratory environment	- Increased thrust phase by 15% with longer poles - Vertical and anterior posterior impulse increased by 17 and 8% with increasing pole length
[38]	N = 7 Gender: 7 m Mean age:/	Roller skiing	Determination of the effects pole length has on energy cost and kinematics in cross-country double poling	Controlled, treadmill	- Increase in propulsive impulse and ground contact time with pole length - Decrease in poling frequency and VO2 with pole length
[8]	N = 11 Gender: 11 m Mean age: 24.0 ± 4.6	Walking	Measurement of the effects pole inertia has on energy cost and EMG activity	Controlled, treadmill	- No difference for VO2 was found for pole length - For heavier poles, biceps brachii activation was higher

## Data Availability

All data are presented in the paper.
